# The Making of a Compound Inflorescence in Tomato and Related Nightshades

**DOI:** 10.1371/journal.pbio.0060288

**Published:** 2008-11-18

**Authors:** Zachary B Lippman, Oded Cohen, John P Alvarez, Mohamad Abu-Abied, Irena Pekker, Ilan Paran, Yuval Eshed, Dani Zamir

**Affiliations:** 1 The Hebrew University of Jerusalem Faculty of Agriculture, Institute of Plant Sciences, Rehovot, Israel; 2 Agricultural Research Organization, The Volcani Center, Bet Dagan, Israel; 3 Department of Plant Sciences, Weizmann Institute of Science, Rehovot, Israel; Max Planck Institute for Developmental Biology, Germany

## Abstract

Variation in the branching of plant inflorescences determines flower number and, consequently, reproductive success and crop yield. Nightshade (Solanaceae) species are models for a widespread, yet poorly understood, program of eudicot growth, where short side branches are initiated upon floral termination. This “sympodial” program produces the few-flowered tomato inflorescence, but the classical mutants *compound inflorescence* (*s*) and *anantha* (*an*) are highly branched, and *s* bears hundreds of flowers. Here we show that *S* and *AN*, which encode a homeobox transcription factor and an F-box protein, respectively, control inflorescence architecture by promoting successive stages in the progression of an inflorescence meristem to floral specification. *S* and *AN* are sequentially expressed during this gradual phase transition, and the loss of either gene delays flower formation, resulting in additional branching. Independently arisen alleles of *s* account for inflorescence variation among domesticated tomatoes, and *an* stimulates branching in pepper plants that normally have solitary flowers. Our results suggest that variation of Solanaceae inflorescences is modulated through temporal changes in the acquisition of floral fate, providing a flexible evolutionary mechanism to elaborate sympodial inflorescence shoots.

## Author Summary

Among the most distinguishing features of plants are the flower-bearing shoots, called inflorescences. Despite a solid understanding of flower development, the molecular mechanisms that control inflorescence architecture remain obscure. We have explored this question in tomato, where mutations in two genes, *ANANTHA* (*AN*) and *COMPOUND INFLORESCENCE* (*S*), transform the well-known tomato “vine” into a highly branched structure with hundreds of flowers. We find that *AN* encodes an F-box protein ortholog of a gene called *UNUSUAL FLORAL ORGANS* that controls the identity of floral organs (petals, sepals, and so on), whereas *S* encodes a transcription factor related to a gene called *WUSCHEL HOMEOBOX 9* that is involved in patterning the embryo within the plant seed. (F-box proteins are known for marking other proteins for degradation, but they can also function in hormone regulation and transcriptional activation) Interestingly, these genes have little or no effect on branching in inflorescences that grow continuously (so-called “indeterminate” shoots), as in *Arabidopsis*. However, we find that transient sequential expression of *S* followed by *AN* promotes branch termination and flower formation in plants where meristem growth ends with inflorescence and flower production (“determinate” shoots). We show that mutant alleles of *s* dramatically increase branch and flower number and have probably been selected for by breeders during modern cultivation. Moreover, the single-flower inflorescence of pepper (a species related to tomato, within the same Solanaceae family) can be converted to a compound inflorescence upon mutating its *AN* ortholog. Our results suggest a new developmental mechanism whereby inflorescence elaboration can be controlled through temporal regulation of floral fate.

## Abstract 3

Plant flower production is largely determined by the number of inflorescences, the branches produced on flower stems. Two genes identified in tomato reveal a new phase transition that may explain the mechanism of evolution of compound inflorescences in the Solanaceae family.

## Introduction

**Figure 1:**
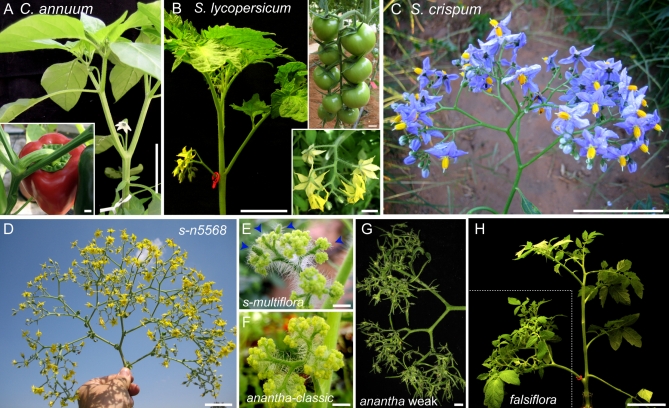
Solanaceae Inflorescences and Mutant Phenotypes (A) Pepper plant showing single-flower inflorescence and mature fruit (inset). (B) Tomato plant and inflorescence (red ring) showing zigzag growth (lower inset) and maturing fruits (upper inset). (C) Branched inflorescence of the species S. crispum. (D) Mutant, highly branched inflorescence of *s* in a mixed genotype with the wild tomato species S. pennellii. (E) Mutant inflorescence of a second allele, *s-multiflora*, having flowers (blue arrows) mixed with cauliflower-like tissue. (F) Mutant, branched *an-classic* inflorescence with cauliflower-like tissue in place of flowers. (G) A weaker *an* allele with sepal and carpelloid tissue. (H) Mutant, branched *fa* inflorescence (dashed box) with leaves in place of flowers. Scale bars in (A, B, C, D, H), 5 cm; in insets (E, F, and G), 1 cm.

A striking manifestation of plant evolution is observed in the diverse branching and patterning of inflorescences, which are the shoots that bear flowers [1,2] Inflorescences are derived from the growth of dome-shaped groups of pluripotent cells called apical meristems. Apical meristems first produce leaves, and upon flowering induction, they produce inflorescence meristems that transition to floral meristems, which produce flowers. Extensive variation in inflorescence complexity is found in the nightshade (Solanaceae) family, where flowering marks the end of main shoot growth, and vegetative aerial growth is renewed from axillary meristems in a perennial growth system known as “sympodial” [3–5]. The simplest Solanaceae inflorescence is a solitary flower, represented by pepper (Capsicum annum) in Figure 1A. Tomato (Solanum lycopersicum), on the other hand, generates a few-flowered inflorescence organized in a zigzag branch (Figure 1B), but there are three classical mutants called *compound inflorescence* (*s*) (Figure 1D and 1E), *anantha* (*an*) (Figure 1F and 1G), and *falsiflora* (*fa*) (Figure 1H) that bear highly branched inflorescences resembling wild Solanaceae species like S. crispum (Figure 1C) [6–8] These similarities suggest that branching complexity may arise from tuning a common underlying developmental program. We set out to begin to unravel the basis of Solanaceae inflorescence diversity using these mutants whose variation ranges from branched inflorescences that produce hundreds of fertile flowers as seen in *s* [6], to the branching shoots of *an* that terminate in cauliflower-like tissue [7], to the leafy inflorescences of *fa*, which is defective in the tomato ortholog of *LEAFY* (*LFY*) [9].

## Results

### Development of Normal and Ramified Inflorescence Types

**Figure 2:**
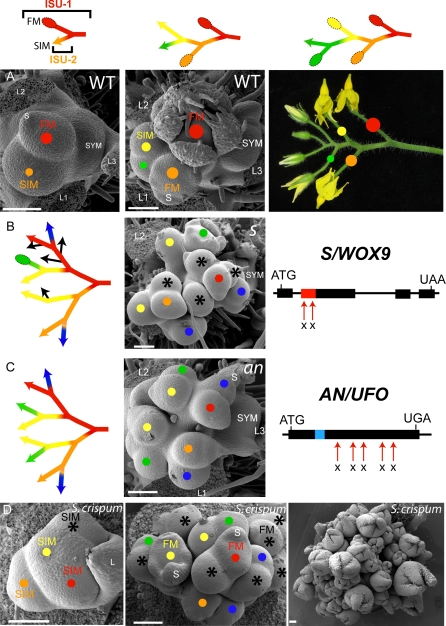
Early Branching Patterns of Normal and Mutant Inflorescences Scanning electron micrographs and schematics of inflorescence development. Schematics reflect sequential inflorescence sympodial units (ISU) each composed of a SIM branch (colored line with arrow) that terminates with a flower (FM, colored oval). Colored circles in micrographs reflect corresponding structures in schematics. (A) Two stages of sympodial inflorescence development and mature zigzag inflorescence. (B) *s* inflorescences develop extra SIMs due to mutations in the ortholog of *WOX9* (red rectangle = homeodomain; mutations marked by red arrows = *s-classic* and *s-multiflora*). Additional SIMs (colored circles) eventually form flowers. Black asteriks reflect asymmetrical development of meristem branches (black arrows in schematics). (C) Strong alleles of *an*, defective in the tomato ortholog of *UFO*, produce extra SIMs instead of flowers (blue rectangle = F-box domain; mutations marked by red arrows). Same color dots and lines reflect SIMs of a similar stage that become branches of the mature inflorescence (see Figure S1 for more details). (D) S. crispum inflorescences showing an *s-*like SIM branching pattern (colored dots/asteriks reflect interpretation of sequential SIM production similar to the convention in (B)). The youngest inflorescence (left) has already produced three SIMs from the leading SIM (red dot), and each of these elaborates further (middle). A later stage inflorescence (right) shows more than 50 maturing flowers. As seen in *s*, the number and position of lateral SIMs derived from leading meristems varies between inflorescences, as does the position of differentiating flowers (see Figure S2 for more details). L = leaf; SYM = sympodial shoot meristem. Scale bars, 100 μm.

The tomato plant is a compound shoot formed from reiterated sympodial shoot units (SYM) that arise from vegetative meristems that produce three leaves before terminating with an inflorescence [10]. The tomato inflorescence is also a compound shoot, which is condensed, consisting of sequential one-nodal inflorescence sympodial units (ISUs) each terminated by a single flower [11]. During early inflorescence development, individual ISUs developed in a progression of two phases. In the first phase, a sympodial inflorescence meristem (SIM), which was distinct from a SYM because it formed within the inflorescence itself, arose and produced a new SIM on its side before differentiating into a floral meristem (FM) in a second phase. These events created the first ISU and the SIM of the second ISU (Figure 2 and Figure S1). This pattern reiterated as subsequent SIMs developed perpendicular to one another, producing a zigzag pattern of flower initiation (Figure 2A). In *an* and *fa* mutants, the primary meristems failed to become flowers, remained indeterminate, and repeatedly initiated secondary SIMs that, themselves, repeatedly produced SIMs (Figure 2 and Figure S1). *s* was more asynchronous, as SIMs eventually transitioned to flowers after producing 2–4 axillary SIMs in a variable, environment-dependent manner (Figure 2 and Figure S1).

Although the branching effects were similar between the three mutants, floral phenotypes were not. Mutants of *fa* were primarily vegetative, producing numerous leaves that developed early as primordia coming off the flanks of meristems (Figure 1H and Figure S1K). Mutants of *an*, on the other hand, produced leaf primordia mixed with other tissue that at maturity resembled modified sepals or bracts (Figure 1F and 1G). It is interesting that *s* mutants maintained the capacity to produce normal flowers, indicating a reduced role in the flower relative to the SIM, although on occasion we observed some leaf-like primordia (Figure S1F). Thus, beyond distinctions in controlling floral organ identity (Figure 1), *s, an*, and *fa* mutants exhibit delayed ISU maturation, resulting in additional branches through the ongoing initiation of lateral SIMs. Notably, SIM branching in diverse Solanaceae is based on an *s-*like program, as seen in early inflorescence development of S. crispum (Figure 2D and Figure S2). This suggests that delays in floral termination (perhaps mediated by *S*, or the genetic pathway that *S* defines) provide a developmental framework for the modulation of sympodial branching in the Solanaceae.

### *compound inflorescence* Encodes a Wuschel-Homeobox Transcription Factor and Is Responsible for a Major Portion of Inflorescence Variation in Domesticated Tomatoes

**Figure 3:**
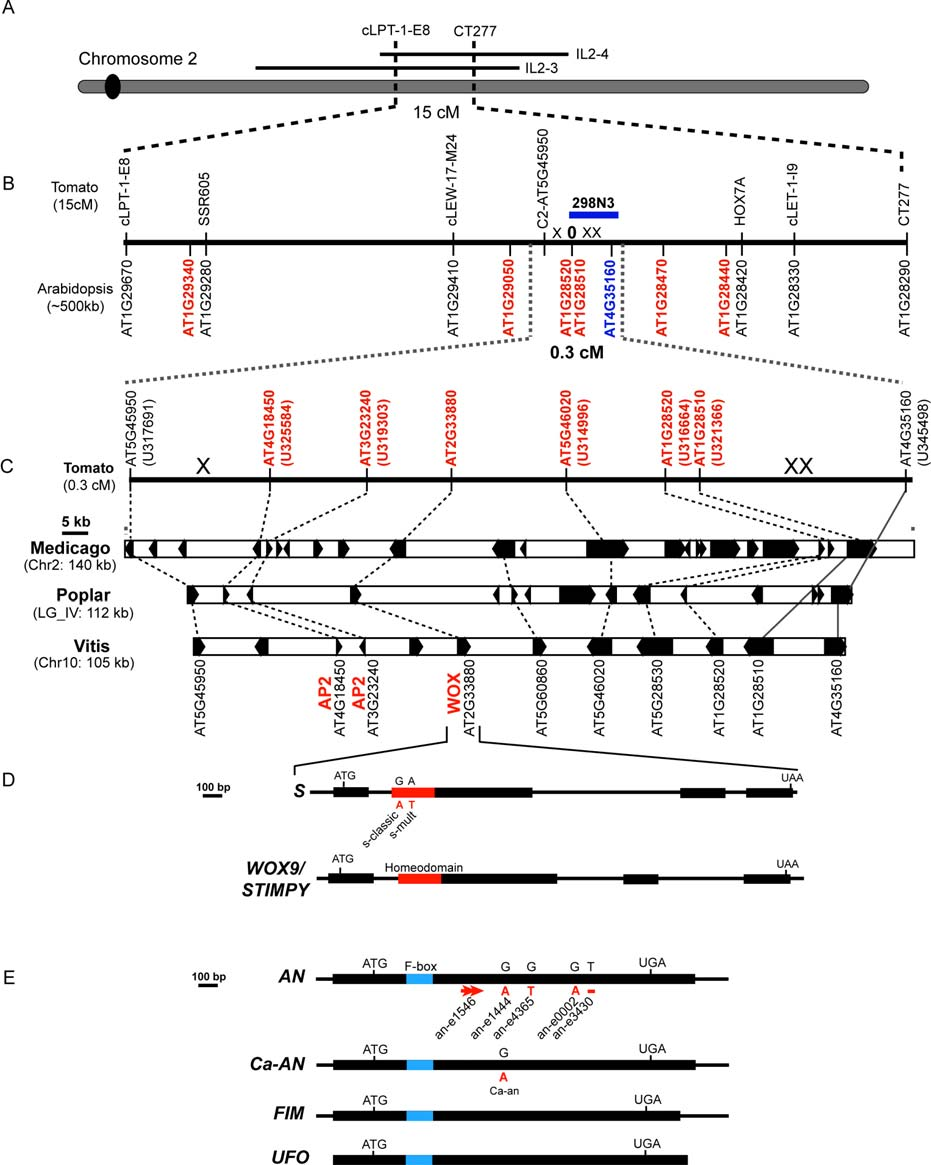
Cloning of the *compound inflorescence* (*s*) and *anantha* (*an*) Genes (A) The 15-cM region of tomato chromosome 2 where *s* was positioned previously showing overlap with the S. pennellii introgression line segments IL2–3/2–4. (B) Tomato markers (black font) spanning the *s* mapping interval and corresponding homologues from *Arabidopsis* showing synteny with genes on chromosome 1. Markers in red were designed according to this synteny, three of which helped to delimit *s* to a 0.3 cM window based on three recombination crossover events (denoted by ‘X'). Two co-segregating markers (‘0') were used to isolate a BAC (blue bar), which provided an additional marker (blue font) to the right of *s*. (C) Physical synteny expanded from 0.3 cM of tomato in four Eudicot species sharing at least seven genes (dashed lines) with three other genes syntenic between a subset of species (solid gray lines). Tomato unigene sequences used to design syntenic PCR markers are in parentheses. Some homologues vary in size, which is due to differences in structural predictions. The three transcription factors (red fonts), two *Apetala2-like* (AP2) and a *Wuschel-homeobox* (WOX) were the primary candidate genes for *s*. (D) Detailed view of the *S* gene structure compared to its *Arabidopsis* homologue *WOX9/STIMPY*. The highly conserved 65–amino acid homeodomain is shown in red, and the two alleles of *s* are indicated with the nucleotides that changed (below the red bar; red font) from wild type (above the red bar; black font). The classic allele of *s* (LA3094; *s-classic*) and *s-mult* (LA0560) had missense mutations altering two invariant amino acids in the homeodomain. Our fast neutron induced allele (*s*-n5568) seems to have suffered a promoter deletion or rearrangement (Figure S3). (E) Detailed view of tomato *AN*, pepper *AN* (*Ca-AN*), and their orthologs from Antirrhinum majus (*FIMBRIATA; FIM*) and *Arabidopsis* (*UNUSUAL FLORAL ORGANS; UFO*). The F-box domain is shown in blue, and five alleles of *an* are indicated with their DNA sequence changes shown below the blue bar. Three strong alleles had premature stop codons due to a 62bp duplication-induced frameshift (*an-*e1546, red arrows), a single base deletion (*an-*e3430, red dash), and a nonsense mutation (*an-*e0002, red font). Three weak alleles (*an*-e1444, *an*-e4365, *Ca-an*) had missense mutations, and *an-classic* (LA0536) suffered from a local rearrangement (Figure S3).

To identify the genes responsible for these phenotypes, *s* and *an* were localized to linked regions of chromosome 2, and *s* was positionally cloned using a remarkable level of multi-genome synteny between the eudicot species poplar (Populus trichocarpa), Barrel Medic (Medicago truncatula), and grape (Vitis vinifera) (Figure 3). Several genes were shared in a short chromosomal segment ranging from 105–140 kb, and aligning these regions revealed three transcription factors: two *AP2-like* genes and a *WUSCHEL-homeobox* (*WOX*) that each co-segregated with *s*. Sequencing of all three genes revealed independent point mutations in the *WOX* gene from two alleles of *s* (*s-classic* and *s-multiflora*), and Southern blot analysis showed chromosomal changes in an additional allele (*s-n5568*), demonstrating that *s* is mutated in this gene (Figure 3 and Figure S3B and S4) (GenBank (http://www.ncbi.nlm.nih.gov/Genbank/) accessions FJ190663 and FJ190664). To our knowledge, this is the first example of gene identification using multi-genome synteny among four eudicot species. Our data suggest that an even greater level of synteny remains to be discovered, and that non-model species will realize similar benefits as more genomes are sequenced.

**Figure 4:**
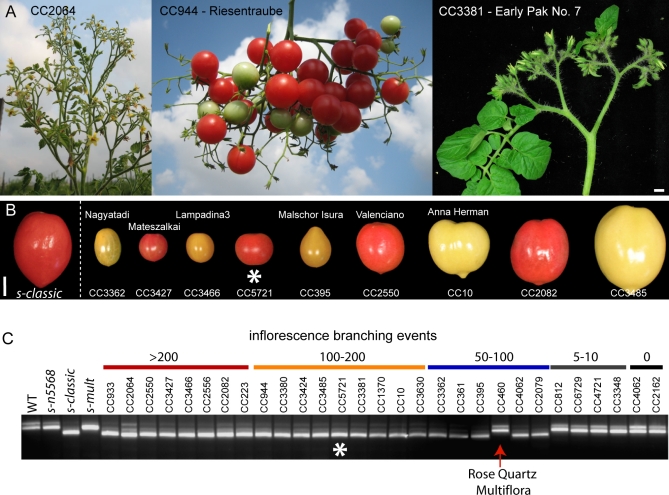
Inflorescence Variation in Domesticated Tomatoes Is Due To Independently Arisen Alleles of *s* The *s-classic* allele was first described 100 years ago as a highly branched variety called “Wonder of Italy,” and garden varieties resembling *s* remain popular for their aesthetic value and prolific fruit production [38]. Six thousand domesticated varieties were screened for inflorescence variation and 23 lines exhibited highly compound inflorescences. Among the 23 lines, at least 15 represented distinct genetic backgrounds based on differences in fruit size, shape, color, and quantitative variation in branch number. (A) Phenotypic variation from three distinct varieties is shown. Core Collection line 2064 (CC2064) was extremely compound as a result of more than 200 branching events, whereas CC944 and CC3381 branched less often, and CC3381 also developed leaves within the inflorescence (B) Variation in fruit size, shape, and color highlighting the different genetic backgrounds of the varieties with compound inflorescences. Varieties with names are indicated. (C) Cleaved amplified polymorphic sequence (CAPS) PCR genotyping assay showing that all except one of 23 varieties with compound inflorescences carry the *s-classic* allele. CC5721 (white asterisk), which carries the identical lesion as *s-classic*, arose independently from a distinct progenitor line (see text for details). Controls were varieties with weak (5–10 branching events) or no branching. Rose Quartz Multiflora was confirmed by complementation test to be an allele of *s*, and arose independently as a result of a genomic rearrangement (Figure S3). Scale bar in (A), 1 cm.

WOX proteins share homology with the meristem maintenance gene *WUSCHEL* and are plant-specific transcription factors [12]. Among 14 *WOX* genes in *Arabidopsis*, *S* is most similar to *WOX9*/*STIMPY* (*STIP*) that functions with *WOX8/STIPL* to regulate embryonic patterning [13]. In contrast, we have found that *S* is a major determinant of inflorescence architecture in tomato. In the context of a European Solanaceae project (Eu-Sol), we established and phenotyped a collection of more than 6000 domesticated tomato varieties for various traits (Materials and Methods; https://www.eu-sol.wur.nl). The power of such a large germplasm resource resides in the fact that extensive natural allelic variation with both qualitative and quantitative effects has been selected and maintained since tomato was first domesticated [14]. Thus, these varieties provide a complement to the stronger, mostly deleterious effects, of alleles derived from artificial mutagenesis [8]. Among the 6,000 tomato lines, we identified 23 accessions with highly branched inflorescences and all were allelic to *s* Surprisingly, 22 of these lines carried the *s*-*classic* allele of the original mutant described 100 years ago, indicating that early breeders were positively selecting this mutant, probably for aesthetic value and fruit production (Figure 4) [6]. However, it was also possible that one or more of these lines arose independently, generating a mutation in the same nucleotide as *s-classic*. To address this question, we sequenced the coding region of all 22 lines and un-branched controls and found that all were identical except CC5721. Interestingly, this line carried four single-nucleotide polymorphisms (SNPs) that were shared with at least one un-branched variety, indicating that the *s* mutation in CC5721 may have arisen independently from a genetically distinct progenitor line (GenBank accessions FJ190665, FJ190666, and FJ190667). Two pieces of evidence lend support to this claim. Firstly, the four SNPs were distributed close (all within 1,000 bp) to the *s* lesion. Secondly, we sequenced a short segment of DNA from the tightly linked bacterial artificial chromosome (BAC) 298N3 and found that CC5721 had six SNPs and a 9–bp insertion-deletion (indel) that distinguished it from the other 21 domesticated types carrying *s-classic* (Figure 3b) (GenBank accessions FJ215691 and FJ215692). Still, in the absence of a geographic distribution of haplotypes, we cannot exclude a remote possibility that CC5721 arose as a result of an intra-genic recombination between *s-classic* and an unbranched variety. Regardless, at least three independently arisen alleles of *s* (*s-classic*, *s-multiflora*, and Rose Quartz Multiflora) are responsible for a major portion of the diversity in tomato inflorescence architecture.

### *anantha* Encodes an F-Box Ortholog of *Arabidopsis* UNUSUAL FLORAL ORGANS (UFO)

The similarity between the phenotypically strong allele *s-multiflora* and strong *an* mutants suggested a functional link in regulating an underlying inflorescence branching program (Figure 1). Furthermore, we created double mutant plants of weak *an* alleles and *s* and found they were phenotypically enhanced to resemble strong *an* (Figure S5). Interestingly, stronger phenotypes were observed for both inflorescence branching and floral identity. Specifically, we found that the sepal and carpelloid tissue of weak *an* mutants became much more meristematic with less organ identity (Figure S5B). In some double mutants, additional leaves formed in the inflorescence, resembling *fa* mutants (unpublished data). This suggests that *S* and *AN* have overlapping roles in inflorescence architecture as well as floral identity. We noted that *an* resembled a Lotus japonicus mutant called *proliferating floral organs* (*pfo*) (Figure 1e) [15]. *PFO* encodes an F-box protein orthologous to *Antirrhinum FIMBRIATA* (*FIM*) and *Arabidopsis UNUSUAL FLORAL ORGANS* (*UFO*) [16], and the tomato ortholog of this gene co-segregated with *an*. Six alleles had mutations in the coding region, revealing that *an* is mutated in the tomato ortholog of *FIM/UFO* (Figures 2C, 3E, and Figure S3A and S6) (GenBank accession FJ190668). The similar inflorescence and floral phenotypes found in *an* and *fa* mutants [17] may, therefore, stem from conserved functional associations of their gene products as described in *Arabidopsis* [18]. However, the relationship between *S* and *AN* was less clear, and their expression patterns were therefore explored.

### *S* and *AN* Are Sequentially Expressed to Promote the Gradual Transition from Inflorescence to Flower Meristem

**Figure 5:**
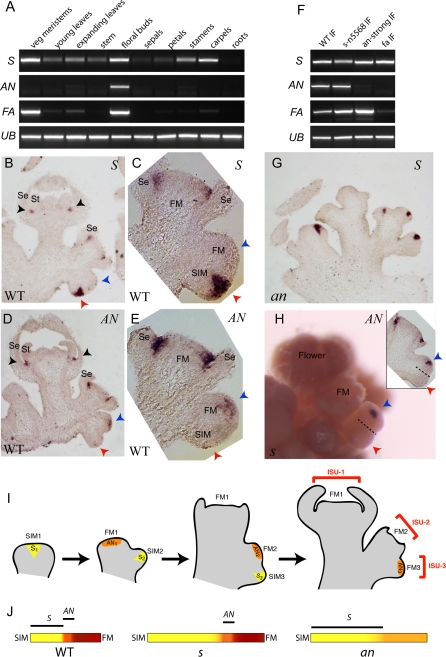
Expression Patterns of Three Inflorescence Architecture Genes (A) RT-PCR of *S*, *AN*, and *FA* transcripts in normal tissues. (B–E) Detection of *S* and *AN* by in situ hybridization. Upper right denotes probe; lower left, genotype. (B) Longitudinal section of a normal inflorescence showing *S* expression in an immature SIM (lower red arrow), but not in a more advanced ISU (upper blue arrow). Weak expression is observed between sepal and petal primordia in flowers (black arrows). (C) Close-up of similarly staged section from (B). (D) Longitudinal section showing *AN* expression in an incipient FM in the upper ISU (blue arrow). Expression is absent in the lower immature SIM (red arrow). *AN* is also expressed between sepal and petal primordial. (E) Close-up of a similarly staged section. (F) RT-PCR of *S*, *AN*, and *FA* in normal and mutant infloresences (IF). (G) Expression of *S* in *an* mutants marking SIMs that remain in a pre-floral state. (H) In situ hybridization with whole-mounted tissue from an *s* mutant; *AN* expression in an advanced SIM (blue arrow), but not in a less mature SIM below (red arrow), matching a similarly staged section (inset). (I) Sequential transient expression of *S* and *AN.* The first SIM (SIM1) expresses *S* (S_1_) and initiates the first phase of the maturation of ISUs. This expression is transient as it turns off prior to activation of *AN* (AN_1_) during the second phase of maturation, which occurs in the same ISU (ISU-1). A newly formed SIM (SIM2) emerges laterally marked by a new round of *S* expression (S_2_), which begins maturation of ISU-2, and this process reiterates to produce a multi-flower inflorescence. (J) Schematic for temporal development (color gradient in bar) over time (position in bar) of a normal (WT) ISU. The SIM (yellow) is short-lived and transitions rapidly (orange) to a FM (red) via activity of *S* (black line above yellow) followed by a short period of expression from *AN* (black line above orange). Mutant ISUs of *s* temporarily stall as SIMs (extended yellow bar) allowing extra SIMs to develop before terminating in FMs. *an* mutants remain in a pre-floral state (extended yellow bar with *S* expression) enabling SIMs to elaborate indefinitely. Se = sepals; St = stamens.

*S* was expressed to varying degrees in all tissues except roots, whereas most *AN* expression was restricted to floral buds, indicating a primary function in inflorescence and flower development. *FA* accumulated predominantly in shoot apices (Figure 5A)[9]. We explored further the expression of *S* and *AN* using in situ hybridization, which revealed temporally distinct patterns during inflorescence development. *S* was expressed in a wedge shape radiating outward from 2–3 cells from the center of immature SIMs (Figure 5B and 5C). This expression initiated shortly after lateral bulging of the SIM and was transient, because it disappeared before floral termination. *AN* expression initiated in incipient FMs shortly after down-regulation of *S. AN* expression was less intense than *S*, and was limited to the upper layers of the rapidly maturing SIM (Figure 5D and 5E). Both genes were reactivated in flower primordia in a ring of cells that marked a boundary domain, first between sepal and petal primordia and later between petals and stamens. These floral expression patterns are consistent with the failure of *an* mutants to initiate normal flowers, suggest a role for *S* in the flower, and likely explain the enhanced developmental and molecular phenotypes that *s* imposes on floral organ identity in weak alleles of *an* (Figure S5). Indeed, double mutants show little or no *an* expression similar to strong *an* mutants alone (Figure S5D).

The expression pattern of *S* suggested that it functioned early in SIM maturation to promote the transition to FM, whereas *AN* operated soon after to provide early FM identity. To test these hypotheses, we examined the expression of *S* and *AN* in *s*, *an*, and *fa* mutants. *S* was expressed in all mutant backgrounds, and, as in wild-type, was detected in younger lateral SIMs of *an* inflorescences (Figure 5F and 5G). This indicates that *an* meristems still reach a pre-floral SIM state. *AN* expression, on the other hand, was undetectable by RT-PCR (reverse-transcriptase PCR) in *fa* mutants, consistent with the proposal that *FA* functions upstream of *AN* [17](Figure 5F). Initial expression of *AN* in *s* mutants was delayed, and subsequently detected in only a small subset of SIMs compared to wild-type. In those meristems expressing *AN*, the signal was deeper and more intense than normal (Figure 5H). In situ hybridization from older inflorescences revealed some meristems lacking *S* and *AN* activity altogether, which we verified by whole-mount in situ hybridization (Figure 5H and unpublished data) This indicates that different meristems are at different phases of ISU maturation, and may also reflect the frequent observation of modified leaves or bracts in older *an* inflorescences if some meristems retain a more vegetative state. Taken together, these expression patterns support a mechanism where *S* and *AN* promote successive stages in the progression of an inflorescence meristem to floral specification through sequential transient activities that gradually promote maturation of SIMs (expressing *S*) to early FMs (expressing *AN*) (Figure 5I). Loss of either gene provides SIMs with an extended period of indeterminacy that facilitates ISU elaboration according to an underlying program of sympodial growth (Figure 5J). Furthermore, the observation that *S* expression is maintained in *an* mutants and vice-versa, and that their expression is restricted to temporally distinct domains, supports the notion that these genes have separate but overlapping functions in the maturation of individual ISUs, consistent with the enhancement of weak *an* alleles by *s* (Figure S5)*.*

### A Branched Inflorescence Is Based on the Gradual Transition of an Inflorescence Meristem to a Floral Meristem

**Figure 6:**
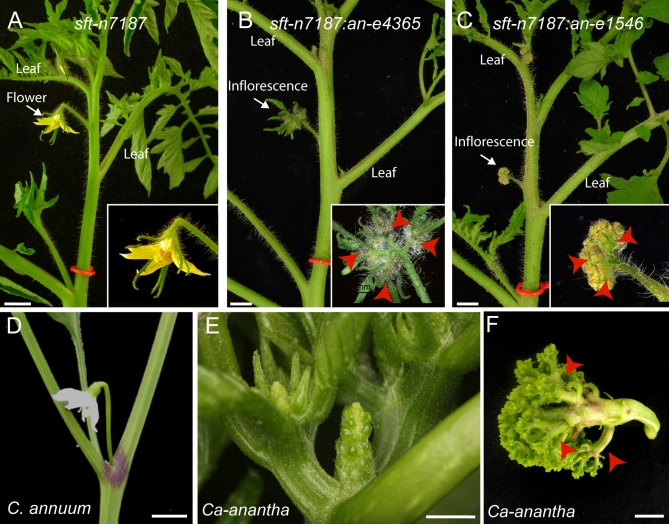
Single-Flower Inflorescence Branching and the *an* Mutant of Pepper (*Ca-an*) (A) Mutant vegetative inflorescence (red ring) of the tomato *sft* mutant showing an isolated flower. (B and C) Double mutant inflorescences (red rings) and flowers (insets) from *sft:an* double mutants with a weak *an* allele (B) and a strong *an* allele (C) showing the conversion of a flower into an inflorescence 3–4 branching events (red arrows). (D) The single flower pepper inflorescence. (E) Mutant, *Ca-an* inflorescence and a highly branched example (F) from a mixed genetic background (three branches are marked with red arrows)

The expression patterns of *S* and *AN* along with their mutant phenotypes lead to a model in which temporal differences in the maturation of a SIM to an FM can regulate the duration of sympodial inflorescence branching. In other words, a slower transition enables more inflorescence branching and vice-versa. This suggests that the SIM phase and the early FM phase of a single flower can each provide a developmental window in which a compound inflorescence can form. We tested this hypothesis genetically by taking advantage of mutants of *single flower truss* (the tomato ortholog of *FT*, which is a major component of florigen), whose inflorescences are indeterminate vegetative shoots with single flowers separated in space by leaves [19] (Figure 6A). In *sft:an* double mutants, we observed that individual flowers became branched inflorescences, though less so than in *an* mutants alone (2–4 versus 20–25 branches at the same age, Figures 1F, 6B, and 6C). By contrast, branching in *sft:s* double mutants resulted in elaboration of the vegetative inflorescence, but normal flowers still formed (Figure S7). Taken together, these results support the proposal that *S* acts earlier within a single inflorescence meristem to regulate sympodial branching, whereas *AN* acts later as FM identity is reached.

Our model suggests that Solanaceae inflorescences with only single flowers may result from rapid termination of the FM and hence elimination of the SIM stage, but that single flower species can still produce branched inflorescences. We addressed this by mutagenizing pepper (*C anuum*), which identified one mutant (called *Ca-an*) that produced an indeterminate shoot instead of a flower. This structure lacked petals and stamens and branched more extensively in a mixed genetic background, resembling tomato *an* mutants (Figure 6E and Figure S8). We sequenced pepper *AN* from *Ca-an* and found a missense mutation from the wild-type progenitor sequence causing a nucleotide change just prior to one of our tomato *an* alleles (*an-*e1444) that co-segregated with the mutant phenotype (Figure S6), indicating that *Ca-an* is mutated in the pepper ortholog of *FIM/UFO* (GenBank accession FJ190669). Like tomato, *Ca-AN* was expressed in a ring of cells flanking developing petals and stamens (Figure S8). Interestingly, *Ca-AN* could not be detected in an earlier inflorescence meristem, which lends support to the idea that pepper has a short SIM phase and progresses rapidly to floral termination. Yet, *Ca-an* mutants revealed a latent potential to branch, indicating that Solanaceae *AN* shares a conserved role in promoting FM determinacy with its orthologs in other species [15,16,20,21]. Of all other known *UFO* mutants, the *pfo* mutant from L. japonicus is most similar to *Ca-an*, with a compact branched structure described as a reiteration of sepals and FMs. Normal L. japonicus produces pairs of flowers in the axils of leaves, and so loss of *UFO* function provides an extended period of indeterminacy to each pair of inflorescence meristems. By contrast, the *stp* mutant of pea generates similar organ defects but produces secondary FMs within the primary flower. Thus, *UFO* has a highly conserved role in floral identity, but its control of inflorescence branching is more species-specific and likely reflects differences in mechanisms of inflorescence meristem initiation. Notably, branching of tomato *an* mutants was more extreme than in *Ca-an* mutants (Figures 1 and 6). This indicates that underlying the tomato SIM phase is a program promoting branching and that the foundation for more complex branching is an inflorescence composed of reiterated SIMs. These data suggest that highly branched species like S. crispum evolved from an ancestral form that resembled tomato, as opposed to pepper (Figure S2).

## Discussion

Our results reveal a genetic foundation for the Solanaceae inflorescence and provide evidence for a possible mechanism that modulates simple and complex inflorescence structures known as “cymes” [1,2] While the generation of a cymose inflorescence through sympodial growth is likely a complex process involving many unknown genetic and environmental factors, we provide a major advance in understanding how cymes may be modified into more complex structures based on elaboration of the ubiquitous ISU shoot system (Figure 2) [5]. This mechanism uses conserved machinery (*AN/UFO* and *FA/LFY*) that regulates inflorescence and flower development in other species [15,16,20–26]. Interestingly, the effects of *UFO* on inflorescence architecture vary considerably, ranging from infrequent replacement of single flowers with secondary inflorescence shoots in Arabidopsis ufo mutants [27], to the production of ectopic flowers in the inflorescences of pea *stp* mutants [20], to the large mass of inflorescence/floral tissue in *pfo* mutants of *L japonicus* [15], and as shown here, the *an* mutant of tomato and pepper. Furthermore, we describe *S/WOX9* as a novel component in the control of inflorescence architecture—a role that was not detected for its *Arabidopsis* ortholog. We also find that the tomato ortholog of *TERMINAL FLOWER1* (*TFL1*) called *SELF PRUNING* (*SP*), which has a major effect on *Arabidopsis* inflorescences [2], is neutral on sympodial inflorescence branching in normal tomato inflorescences (unpublished data), and exhibits indirect effects on *s* inflorescence branching (Table S1). These differences may originate from the evolution of distinct growth habits. Branching complexity in sympodial species relies on termination of inflorescence meristems through the transition of a SIM to an FM. We suggest that a transient expression of *S* followed by *AN* was co-opted in *Solanaceae* sympodial development to boost two phases of sympodial meristem growth in this specialized shoot, both of which can potentiate branching (Figure 3I). In monopodial dicot species such as *Arabidopsis* or *Antirrhinum*, the inflorescence meristem produces no comparable SIMs, being indeterminate and generating lateral single flowers. This indeterminacy may explain why *WOX9*, by itself, is dispensable for inflorescence development [28,29]. Indeed, inflorescence ramification in monopodial dicot plants is more often stimulated through identity change [16,30,31], which could also explain some of the branching effects observed in *Ca-an* (Figure 6)

Thus, while the evolutionary diversification of plant inflorescence architecture is united under a common developmental theme [2], plants with different growth habits use related as well as distinct developmental modules to regulate branching [32]. We propose that Solanaceae inflorescence variation is based on controlling sympodial branching through temporal changes in the acquisition of floral fate, which is most flexible within the SIM phase. Short delays in the activation of genes like *S* (or as-yet-undiscovered other genes in the *S* pathway) followed by an abrupt switch to floral termination may explain the evolution and quantitative variation of compound inflorescences in the genus *Solanum* (Figure S9), as well as in other sympodial species, like trees [5]. Such a mechanism would provide a flexible way to guarantee the production and simultaneous maturation of large numbers of flowers, thereby ensuring a crucial aspect of reproductive success and perhaps providing a new tool for the manipulation of crop yields.

## Materials and Methods

### Plant material and gene cloning.

Classic alleles of *s* (*s-classic* LA3094), *an* (LA0536), and *fa* (LA0854), and those of representative wild tomato species were gifts from the C. M. Rick Center (Davis, California; http://tgrc.ucdavis.edu). An additional allele of *s* (LA0560; *s-multiflora*, C. M Rick Center) was verified by complementation test. A third *s* allele and six additional *an* alleles were identified as inflorescence mutants in a screen of a tomato mutant library [8]. Wild tomato species were gifts from the C. M. Rick Center. Wild tomato species, such as S. lycopersicoides, can be difficult to grow and maintain until flowering and only two representative plants were available for phenotypic analyses, but inflorescence complexity within each plant was uniform throughout. More distantly related Solanum species were gifts from the Botanical and Experimental Garden at Nijmegen, The Netherlands. Up to three representative plants were used for phenotypic analyses. The ∼6,000 domesticated tomato varieties were collected from various public and private germplasm sources. All plants were grown in greenhouses under natural light or in agricultural field conditions in Israel using standard irrigation and fertilization regimes.

### *compound inflorescence* (*s*)

The *s* mutant was originally mapped on the long arm of chromosome 2, and verified using 20 mutants selected from an F2 population derived from a cross with the wild species S. pimpinellifolium (LA1589). This positioned *s* in the region overlapping introgression lines IL2–3/2–4 on the tomato introgression line map [33]. A larger mapping population was generated by crossing *s-*n5568 with the wild tomato species S. pennellii (LA0716). F1 hybrid plants were self-fertilized to produce a mapping population of 5,000 F2 plants. Five hundred individual *s* mutant plants were scored with CAPS-PCR markers from the most current tomato genetic map (Solanaceae Genomics Network at http://www.sgn.cornell.edu), focusing on the region of IL2–3/2–4. Additional markers surrounding the tightly linked *CNR* locus were provided by K. Manning [34]. Marker density was improved using conserved synteny identified between seven markers in a 15-cM window in tomato and a 500-kb segment of *Arabidopsis* chromosome 1 (marker information available upon request). Two co-segregating markers (0 recombinant chromosomes out of 1,000 gametes) were used to isolate a BAC from a S. lycopersicum HindIII library kindly provided by J. J. Giovannoni and J. VanEck at Cornell University (Ithaca, New York). DNA fragments from three independent restriction enzyme digestions of a single BAC clone were sub-cloned into TOPO TA cloning vectors (Invitrogen) for shotgun sequencing. Fragments containing genes were annotated using BLASTX against the *Arabidopsis* protein database and used to search other genomes for additional synteny. Sequences from four tightly linked markers (Figure 3) were used in a BLASTN or TBLASTX search against genomes of *P trichocarpa*, *M truncatula*, and *V vinifera*. Genes in syntenic regions ranging from 110–140 kb were aligned manually and searched for candidate genes, which identified the *Apetala-2* (*AP2*) and *Wuschel-homoebox* (*WOX*) transcription factors. A tomato-specific *WOX* marker was produced by degenerate PCR based on conserved regions in the *WOX* from these three species and an EST from Petunia hybrida (accession number EB174485). Transcript ends were determined by rapid amplification of cDNA ends (RACE) (Sambrook) using total RNA isolated from young inflorescences with TriReagent (Sigma-Genosys). DNA from *s-like* varieties with compound inflorescences from the Core Collection was PCR amplified with gene-specific primers and used in a CAPS-PCR assay diagnostic of *s-classic*.

The expressivity of the *s* phenotype is affected by genetic background, which became evident when phenotyping 22 domesticated varieties each carrying the *s-classic* allele, but varying in many phenotypic characters, including branching. Modifiers are responsible for these differences, which may or may not have a functional relationship to *S*. Furthermore, it is well-documented that sympodial shoot growth in tomato is highly sensitive to light intensity, which could also contribute to quantitative variation between accessions. On occasion, modestly branched accessions were observed that produced only 2–4 additional branches compared to normal, which, if not allelic to *s*, could potentially modify (enhance) the *s* phenotype. Yet, the majority of extreme branching variation was due solely to changes in *S* function, indicated by normal segregation of families segregating for each *s* allele in a common genetic background (cv. M82). Thus, differences in phenotypic strength, as seen in *s-multiflora*, result from modifier loci, but these are much weaker in their effects compared to *s* mutations.

### *anantha* (*an*)

The *an* mutant was originally mapped to the long arm of chromosome 2, and subsequently positioned in the region overlapping IL2–3/2–4/2–5. The phenotype of the *pfo* mutant in *L japonicus* resembled weak alleles of *an* and led us to search for the tomato ortholog of *FIM/UFO.* A single EST (SGN-U341425) with homology to *FIM/UFO* was used to generate a CAPS-PCR marker that mapped to the same region as *an* (http://www.sgn.cornell.edu). DNA from six EMS alleles was amplified using gene-specific primers and sequenced directly, which identified five independent mutations. The central portion of coding sequence of the *an-classic* allele could not be amplified by PCR, suggesting a structural change or large insertion (unpublished data). This rearrangement in the *an-classic* allele was verified using DNA Southern blot hybridizations (Figure S3) according to established protocols.

### Pepper (Capsicum annuum) and the pepper *anantha* mutant (*Ca-an*)

An EMS mutagenesis of the pepper variety Maor was performed according to a protocol for tomato seeds [8]. Among 1,500 M2 families of pepper, one inflorescence mutant was identified based on phenotypic similarity to weak alleles of tomato *an* This new mutant was first mapped by restriction fragment length polymorphism (RFLP) analysis to a region of chromosome 2 in pepper that is syntenic with tomato chromosome 2 where anantha was positioned previously. DNA from the mutant was isolated and sequenced using primers designed from the tomato gene. Co-segregation of the mutation with the pepper *an* phenotype was verified in an F2 population of approximately 100 plants, and the mutation was found to be derived from the Maor variety progenitor sequence. Our allele changes a nearly invariant glycine among F-box proteins into a charged amino acid, glutamic acid (see Figure S6). This glycine is the second amino acid in a short stretch of ∼10 highly conserved amino acids in UFO orthologs for which at least one mutant allele is available in *Arabidopsis*, pea, tomato, and now pepper. Thus, this region is a hot spot for mutations that give very similar floral phenotypes in multiple species.

### Phenotypic and expression analyses.

Developmental and morphological analyses on single and double mutants were performed on alleles originating from the tomato cultivar M82. M82 lines were either mutant or wild type for the gene *SELF PRUNING* (*SP*), which had only modest effects on inflorescence phenotypes in *s* or *an* that could be attributed to changes in the length of sympodial units—a phenotype regulated by *SP*. Single and double mutants of *sft* were the allele *sft-7187* [19]. Mutants of *fa* were in the background of Rheinlands Ruhm. Branching events were counted on two independent inflorescences from Core Collection varieties containing *s-classic* and non-*s* controls. For SEM, immature inflorescences from sympodial shoots were dissected and processed through an EtOH series, critical-point dried, and coated with gold particles for microscope analysis on a Philips XL30 ESEM FEG. RT-PCR was performed using a One-Step RT-PCR kit (Qiagen) on total RNA isolated by TriReagent (Sigma-Genosys) according to the manufacturers' protocols. Primer sequences are available upon request. Tissues for in situ hybridization were dissected and fixed according to standard protocols [35]. In vitro transcribed RNA probes were generated from 5′ partial (*S*) or full-length (*AN*) cDNA clones and transcripts were detected using standard in situ hybridization techniques. Whole-mount in situ hybridization was performed as described [36], using the same probes.

## Supporting Information

Figure S1 Temporal Progression of Early Branching Patterns in Normal and Mutant Inflorescences Scanning electron micrographs of inflorescence development and corresponding schematics are shown. Colored lines and ovals in schematics reflect individual inflorescence sympodial units (ISUs) composed of a SIM branch that terminates in a flower (FM). Identically colored circles in micrographs reflect ISUs in the schematics.
(A) Normal inflorescences give rise to sequential SIMs that rapidly become flowers, resulting in a zigzag mature inflorescence (B).
(C–F) *s* inflorescences are delayed in flower formation, causing SIMs to develop asynchronously, but on average, 2–4 additional SIMs were generated before floral termination of each leading SIM (colored circles). Flowers that form vary in number and position between inflorescences. Black asterisks (black lines in schematics) reflect asymmetrical development of additional meristem branches. Despite this asymmetry, relatively uniform branching patterns emerge in mature inflorescences (G).
(H) Strong alleles of *an* fail to form flowers, and instead produce secondary SIMs that develop perpendicular to previous meristems, which is reflected in the branching pattern of mature mutant inflorescences (I). Same color dots and lines reflect SIMs of a similar stage that become branches in mature inflorescences.
(J) SIM proliferation in a more advanced *an* inflorescence.
(K) Like in *an*, the first two meristems of *fa* fail to form flowers and produce secondary SIMs that become branches in mature inflorescences (L).
(M) Meristem proliferation in a more advanced *fa* inflorescence. Mature inflorescences in (B), (G), (I), and (L) were flattened to capture all branches. L = leaf; SYM = sympodial shoot meristem; SIM = sympodial inflorescence meristem; FM = flower meristem. Scale bars indicate 100 μm; Mature inflorescences, 1 cm.
(2.55 MB JPG)

Figure S2 Temporal Progression of Early Inflorescence Branching Patterns in the Wild Species S. crispum
Scanning electron micrographs (numbered 1–8) present a developmental range of individual inflorescences from the transition to flowering (1) to multi-flower differentiation (7,8). Colored circles in micrographs reflect one possible interpretation of the sequential development of individual SIMs according to the convention used in Figure 2. The overall pattern of SIM production is variable where SIMs present in some inflorescences are absent in others, making other interpretations possible. Like *s*, each SIM produced 2–4 additional SIMs and this elaboration was also asynchronous (black asterisks). As well, the position of the first differentiating flower varied between inflorescences as seen for *s* in Fig. S1. Later stage inflorescences were too complex to be marked. At maturity, S. crispum branched an average of 25 times and produced more than 100 flowers per inflorescence.
(1.64 MB JPG)

Figure S3 Genomic Rearrangements of Alleles of *s* and *an*
(A) DNA Southern blot showing genomic changes in *an-classic* (LA0536). Genomic DNA from wild type (WT), a mix of WT and heterozygous (HET), and mutant (*an*) plants from a segregating family was digested with four restriction enzymes and probed with the full length *AN* gene. Band shifts were observed in *an*, but not in WT alone, and WT/HET samples showed heterozygosity. One explanation for the *an-classic* allele is a transposon insertion within the gene, which would explain the increase in size of the mutant band. Consistent with this idea, we were unable to PCR amplify the central portion of the gene (unpublished data), indicating a chromosomal change in the coding sequence. However, other types of rearrangements could also explain this result, which we did not explore.
(B) DNA Southern blot showing genomic changes in *s-*n5568 (s) and Rose Quartz Multiflora (RQM). For both mutants, we were unable to find mutations by sequence analysis, and we were unable to PCR amplify the 3′ prime end of *S* in RQM mutants, suggesting genomic changes for both alleles. Genomic DNA from wild-type (WT, domesticated cultivar, M82) and mutant plants of *s*-n5568 (M82 background) and RQM (unknown background) was digested with five restriction enzymes and hybridized with a probe corresponding to the 5′ portion of *S* gene. For *s*, a lower band shift (red arrows) was observed for two enzymes, suggesting a large deletion. *s-*n5568 was produced in a fast neutron mutagenesis, which is consistent with this idea; however, the deletion would have to reside upstream or downstream of the coding sequence, because we were still able to amplify this allele by PCR, and we still detect transcripts by RT-PCR and in situ hybridization, albeit weaker than in wild type by in situ hybridization (unpublished data). Three enzymes revealed band shifts (blue arrows) in RQM relative to WT. Such a high frequency of intra-specific polymorphisms by DNA blot is rare in domesticated tomato varieties [37], suggesting these changes are associated with the mutant phenotype. A number of rearrangements are possible, which we did not explore further.
(1.03 MB JPG)

Figure S4 Multiple Alignment of *S* with Highly Related *WOX9* Proteins Highly conserved amino acids are shaded in black, and different amino acids of the same group are shaded in gray. The consensus sequence is shown below the alignment. Dashes denote gaps that were introduced to optimize the alignment. The homeodomain is boxed. The mutations for two missense alleles of *s* (*s-classic* and *s-multiflora*) causing amino acid changes of invariant residues in the homeodomain are indicated above the alignment in red font. Sl-S= *Solanum lycopersicum S*; Ph-WOX9A= *Petunia x hybrida* WOX9A (GenBank accession number EB174497); Ph-WOX9B= *Petunia x hybrida* WOX9B (accession number EB174485); Pt-WOX9A= Populus tricocarpa (accession number CAJ84153); Pt-WOX9B= Populus tricocarpa (genome protein ID: 555728); Mt-WOX9= Medicago truncatula (accession number ABN09121); Vv-WOX9= Vitis vinifera (accession number CAO66373); At-WOX9-STIP= Arabidopsis thaliana Stimpy (accession number NP_180944); At-WOX8-STIPL= Arabidopsis thaliana Stimpy-like (accession number NP_199410).
(2.73 MB JPG)

Figure S5 Enhanced Inflorescence and Floral Phenotypes in Double Mutants between *s* and Weak Alleles of *an*
(A) Young branching inflorescence of a weak *an* allele (*an-*e4365) having sepals and carpelloid tissue, but lacking petals and stamens.
(B) Double mutant of *an-*e4365 with *s-*n5568 showing an enhanced inflorescence phenotype having stronger floral organ defects resembling strong alleles of *an*, like *an-*e1546 shown in (C).
(D) RT-PCR of *AN* expression on single and double mutants. The weak allele *an-*e4365 shows a modest reduction in *AN* expression relative to WT inflorescences. The strong allele *an-*e1546 has little or no *AN* expression, and a similar loss of expression is recapitulated in the enhanced double mutants *s-*n5568:*an*-e4365. This suggests either that *S* has a transcriptional regulatory role on *AN* or that the double mutant arrests at a developmental stage lacking early FM identity, and therefore does not express *AN* at substantial levels.
(1.16 MB JPG)

Figure S6 Multiple Alignment of *AN* with Highly Related F-Box Proteins Highly conserved amino acids are shaded in black, and different amino acids of the same group are shaded in gray. The consensus sequence is shown below the alignment. Dashes denote gaps that were introduced to optimize the alignment. The F-box domain is boxed. The mutations for five alleles of *an* causing frame-shifts or amino acid changes are indicated above the alignment in red font. Red arrows for *an-*e1546 indicate the site of a tandem duplication. The pepper *an* (*Ca-an*) mutation is shown in blue below the alignment preceding *an-*e1444. Am-FIM= Antirrhinum majus FIMBRIATA (FIM, accession number S71192); At-UFO= Arabidopsis thaliana UNUSUAL FLORAL ORGANS (UFO, accession number X89224); Lj-UFO= Lotus japonicus Proliferating floral organs (PFO, accession number AAN87351); Ps-UFO= *Pisum sativum Stamina pistilloida* (Stp, accession number AF004843); Impatiens balsamina FIMBRIATA (Imp-FIM, accession number AF047392).
(2.28 MB JPG)

Figure S7 Inflorescence Branching Phenotypes in Double Mutants of *s* and *sft*
Double mutants between *s* and *sft* convert the single indeterminate vegetative inflorescence shoot of *sft* mutants to a highly branched vegetative inflorescence (red ring, white arrow) with dispersed single flowers (yellow arrowheads). SIM elaboration from a young developmental stage (inset) shows branches composed of leaflets and floral buds (red arrowheads) developing behind a single terminal flower (yellow arrow). These are the vegetative inflorescence branches that contribute to the architecture of mature double mutant inflorescences. Scale bars, 10 cm plant; 100 μm, inset.
(1.95 MB JPG)

Figure S8 Early Development and *AN* Expression in Inflorescences of Normal Pepper and *Ca-an* Mutants (A–D) Scanning electron micrographs showing two sympodial shoots (SYM) and distinct inflorescence stages from normal (A and C) and *Ca-an* mutants (B and D). Normal pepper produces a single floral meristem (FM, red dot) flanked by two SYMs, which are composed of two leaves and a single flower that terminates rapidly. Sympodial shoots arise reiteratively in the axils of each sympodial leaf and are released from apical dominance asymmetrically after floral termination, which is reflected in the slightly different developmental stages. *Ca-an* mutants produce an indeterminate shoot (asterisk) that repeatedly gives off lateral organs, which are, perhaps, modified sepals. Branching is infrequent at this early stage, and occurs more often in mature inflorescences or in the presence of modifiers, as shown in Figure 6F.
(E and F) Detection of pepper *Ca-AN* expression by in situ hybridization. (E) Longitudinal section from a young inflorescence showing expression in the developing FM interior to sepal primordia (black arrows), but not in flanking SYMs (red arrows).
(F) A later stage inflorescence with early stage FMs (left and right). The earliest expression of *Ca-AN* is observed in a ring (black arrows) between sepals (Se) and incipient petals. St= stamen; L= leaf; SYM= sympodial meristem; FM= floral meristem. Scale bars, 100 μm.
(3.46 MB JPG)

Figure S9 Inflorescence Variation in the Genus *Solanum*
Quantification of inflorescence branching events from five species in the genus *Solanum* revealing that inflorescence branching varies widely between species and may have evolved multiple times. Numbers in parentheses indicate average number of branching events in each inflorescence.
(674 KB JPG)

Table S1 The Tomato Ortholog of *TFL1*, called *SP*, Does Not Directly Influence Inflorescence Architecture Comparison of inflorescence branching events in three consecutive inflorescences from s mutant plants carrying a functional (SP+) or mutant (sp–) copy of *SP*, the tomato ortholog of *TFL1.* The first inflorescence after the transition to flowering (primary IF) showed a modest decrease in branching in *s:sp* double mutants (highlighted in gray). Interestingly, this effect was reversed in the two following inflorescences of the first (1^st^ SYM IF) and second (2^nd^ SYM IF) sympodial shoots of *s:sp* plants, which each showed more branching events than in *s* mutants alone (highlighted in gray). These effects are primarily indirect effects of *sp* mutants, whose primary change is on sympodial unit length (i.e., the number of leaves between sympodial units), which decreases progressively as *sp* plants mature [10]. This allows the initiation of both the first and second SYM IF in *s:sp* to occur earlier and, therefore, undergo more branching events compared to correspondingly younger inflorescences in *s* mutants alone. Eventually these younger inflorescences underwent a similar number of branching events, although more variation was introduced as inflorescences aged (unpublished data).
(50 KB DOC)

## Abbreviations

*AN*: *ANANTHA*
BAC: bacterial artificial chromosome
FM: floral meristem
ISU: inflorescence sympodial units
*S*: *COMPOUND INFLORESCENCE*
SIM: sympodial inflorescence meristem
SYM: sympodial shoot meristem

## Note Added in Proof

While this article was under review, additional reports [39,40] have indicated that the orthologs of *S* and *AN* in Petunia hybrida (also a Solanaceae species), called *EVERGREEN* (*EVG*) and *DOUBLE TOP* (*DOT*), have similar functions within inflorescence meristems.
