# Supplementary figures and images for: The Making of a Compound Inflorescence in Tomato and Related Nightshades
Source: PLoS Biol. 2008 Nov 18;6(11):e288. doi: 10.1371/journal.pbio.0060288 (PMC2586368; doi:10.1371/journal.pbio.0060288)

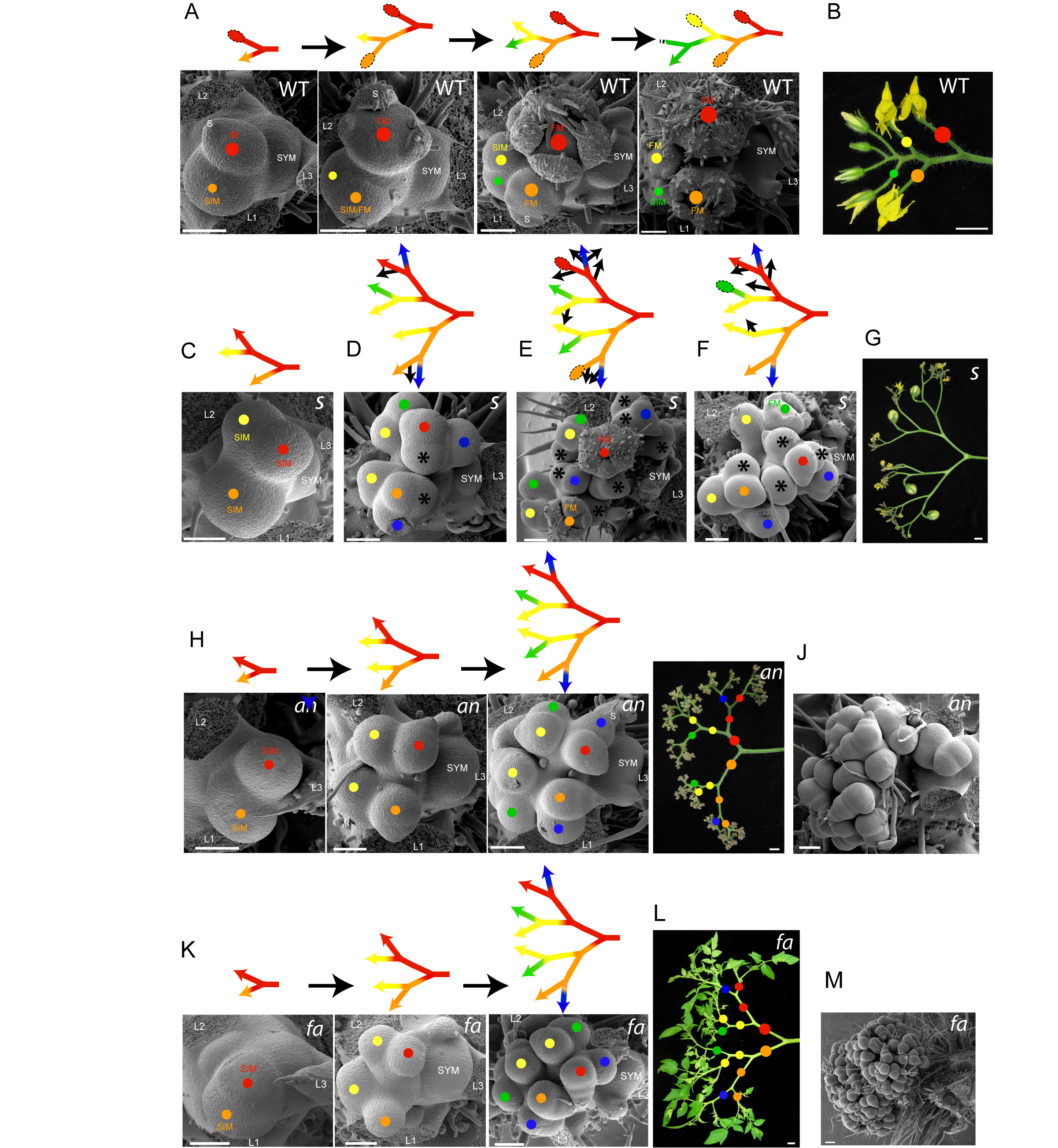

Supplement: Figure S1 — Scanning electron micrographs of inflorescence development and corresponding schematics are shown. Colored lines and ovals in schematics reflect individual inflorescence sympodial units (ISUs) composed of a SIM branch that terminates in a flower (FM). Identically colored circles in micrographs reflect ISUs in the schematics. (A) Normal inflorescences give rise to sequential SIMs that rapidly become flowers, resulting in a zigzag mature inflorescence (B). (C–F) s inflorescences are delayed in flower formation, causing SIMs to develop asynchronously, but on average, 2–4 additional SIMs were generated before floral termination of each leading SIM (colored circles). Flowers that form vary in number and position between inflorescences. Black asterisks (black lines in schematics) reflect asymmetrical development of additional meristem branches. Despite this asymmetry, relatively uniform branching patterns emerge in mature inflorescences (G). (H) Strong alleles of an fail to form flowers, and instead produce secondary SIMs that develop perpendicular to previous meristems, which is reflected in the branching pattern of mature mutant inflorescences (I). Same color dots and lines reflect SIMs of a similar stage that become branches in mature inflorescences. (J) SIM proliferation in a more advanced an inflorescence. (K) Like in an, the first two meristems of fa fail to form flowers and produce secondary SIMs that become branches in mature inflorescences (L). (M) Meristem proliferation in a more advanced fa inflorescence. Mature inflorescences in (B), (G), (I), and (L) were flattened to capture all branches. L = leaf; SYM = sympodial shoot meristem; SIM = sympodial inflorescence meristem; FM = flower meristem. Scale bars indicate 100 μm; Mature inflorescences, 1 cm. (2.55 MB JPG) [file pbio.0060288.sg001.jpg]

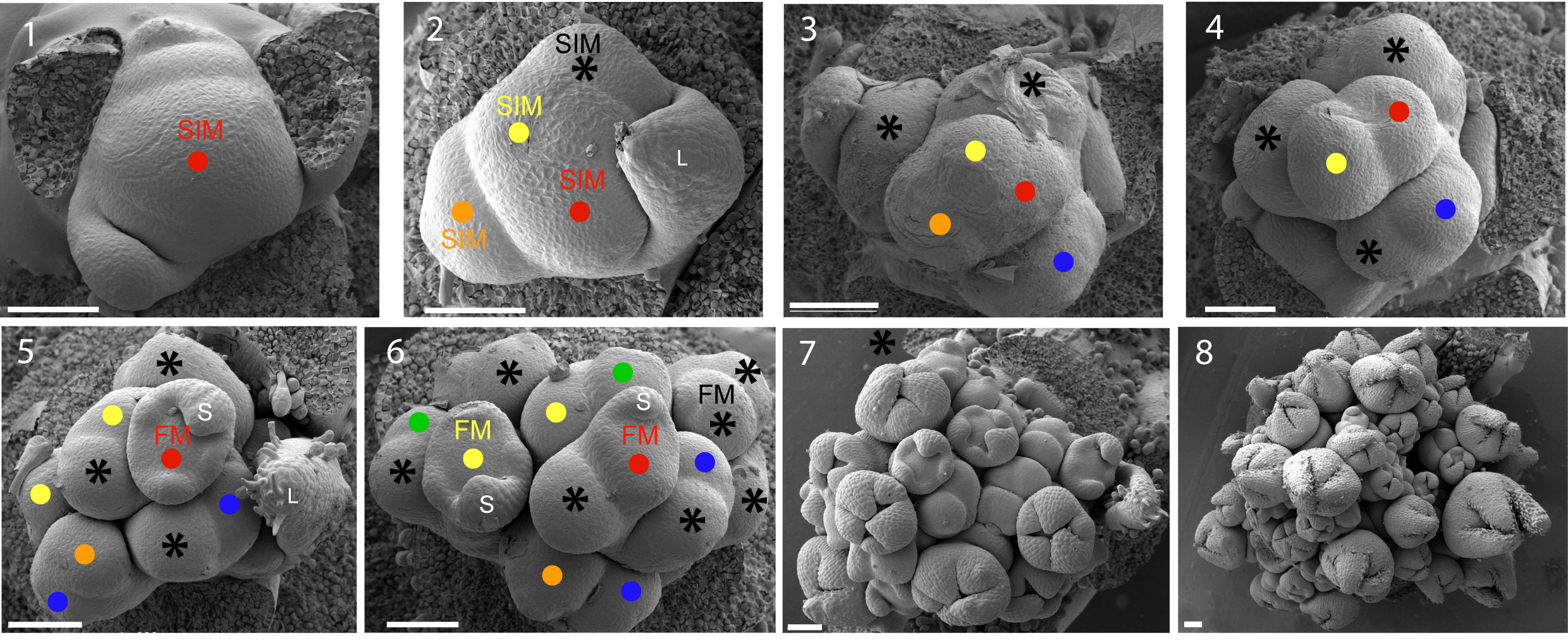

Supplement: Figure S2 — Scanning electron micrographs (numbered 1–8) present a developmental range of individual inflorescences from the transition to flowering (1) to multi-flower differentiation (7,8). Colored circles in micrographs reflect one possible interpretation of the sequential development of individual SIMs according to the convention used in Figure 2. The overall pattern of SIM production is variable where SIMs present in some inflorescences are absent in others, making other interpretations possible. Like s, each SIM produced 2–4 additional SIMs and this elaboration was also asynchronous (black asterisks). As well, the position of the first differentiating flower varied between inflorescences as seen for s in Fig. S1. Later stage inflorescences were too complex to be marked. At maturity, S. crispum branched an average of 25 times and produced more than 100 flowers per inflorescence. (1.64 MB JPG) [file pbio.0060288.sg002.jpg]

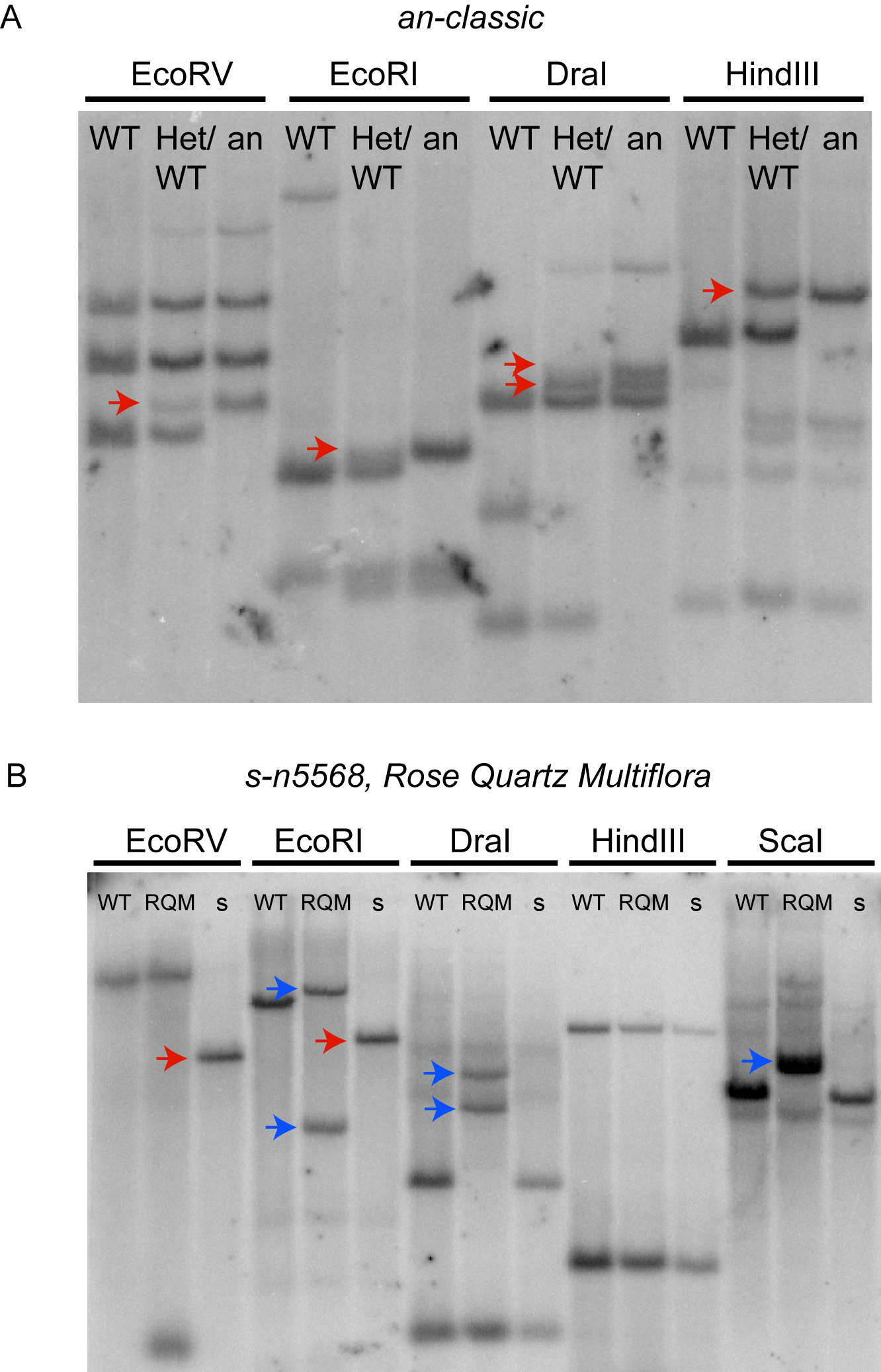

Supplement: Figure S3 — (A) DNA Southern blot showing genomic changes in an-classic (LA0536). Genomic DNA from wild type (WT), a mix of WT and heterozygous (HET), and mutant (an) plants from a segregating family was digested with four restriction enzymes and probed with the full length AN gene. Band shifts were observed in an, but not in WT alone, and WT/HET samples showed heterozygosity. One explanation for the an-classic allele is a transposon insertion within the gene, which would explain the increase in size of the mutant band. Consistent with this idea, we were unable to PCR amplify the central portion of the gene (unpublished data), indicating a chromosomal change in the coding sequence. However, other types of rearrangements could also explain this result, which we did not explore. (B) DNA Southern blot showing genomic changes in s-n5568 (s) and Rose Quartz Multiflora (RQM). For both mutants, we were unable to find mutations by sequence analysis, and we were unable to PCR amplify the 3′ prime end of S in RQM mutants, suggesting genomic changes for both alleles. Genomic DNA from wild-type (WT, domesticated cultivar, M82) and mutant plants of s-n5568 (M82 background) and RQM (unknown background) was digested with five restriction enzymes and hybridized with a probe corresponding to the 5′ portion of S gene. For s, a lower band shift (red arrows) was observed for two enzymes, suggesting a large deletion. s-n5568 was produced in a fast neutron mutagenesis, which is consistent with this idea; however, the deletion would have to reside upstream or downstream of the coding sequence, because we were still able to amplify this allele by PCR, and we still detect transcripts by RT-PCR and in situ hybridization, albeit weaker than in wild type by in situ hybridization (unpublished data). Three enzymes revealed band shifts (blue arrows) in RQM relative to WT. Such a high frequency of intra-specific polymorphisms by DNA blot is rare in domesticated tomato varieties [37], suggesting these changes [file pbio.0060288.sg003.jpg]

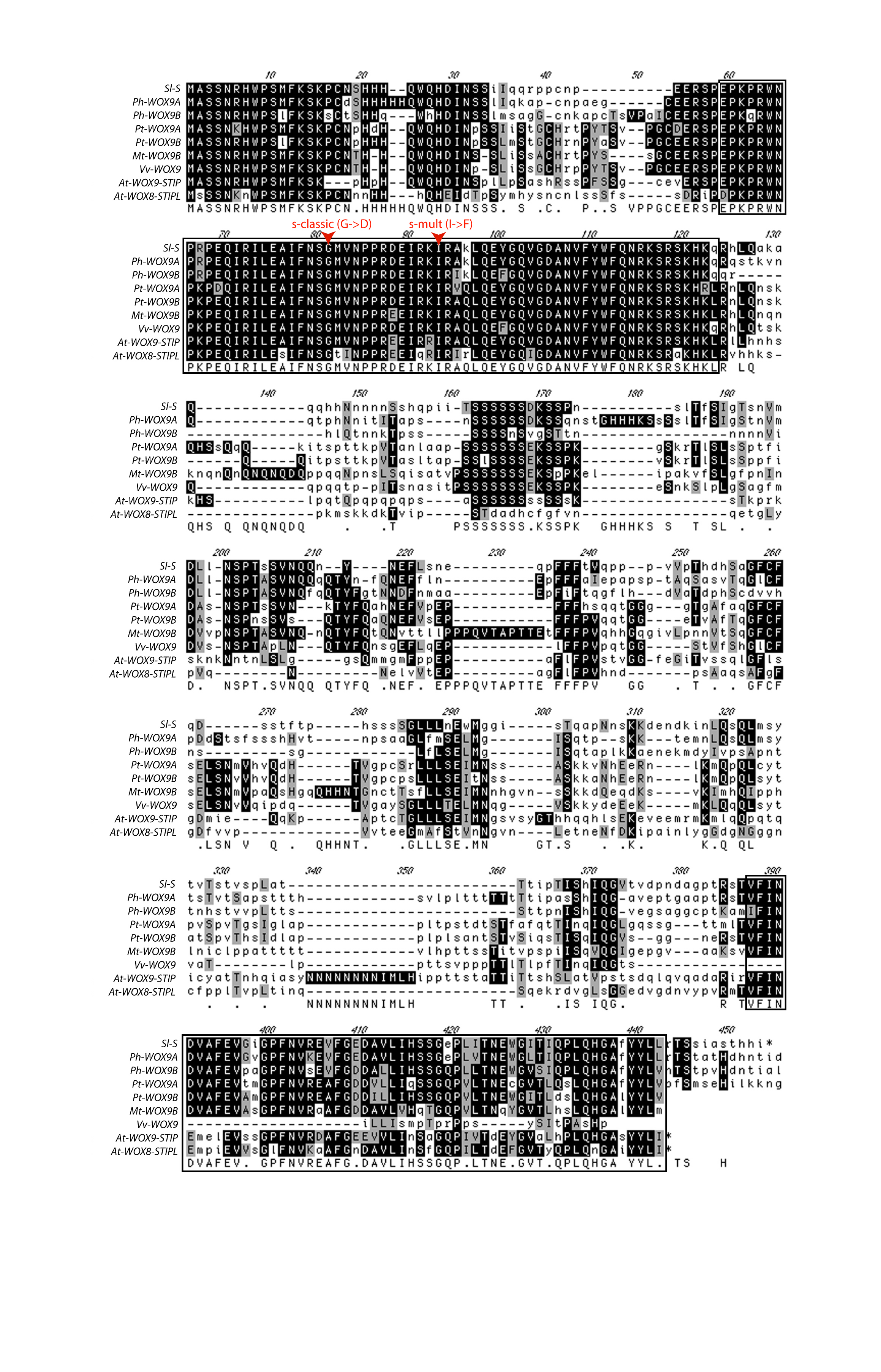

Supplement: Figure S4 — Highly conserved amino acids are shaded in black, and different amino acids of the same group are shaded in gray. The consensus sequence is shown below the alignment. Dashes denote gaps that were introduced to optimize the alignment. The homeodomain is boxed. The mutations for two missense alleles of s (s-classic and s-multiflora) causing amino acid changes of invariant residues in the homeodomain are indicated above the alignment in red font. Sl-S= Solanum lycopersicum S; Ph-WOX9A= Petunia x hybrida WOX9A (GenBank accession number EB174497); Ph-WOX9B= Petunia x hybrida WOX9B (accession number EB174485); Pt-WOX9A= Populus tricocarpa (accession number CAJ84153); Pt-WOX9B= Populus tricocarpa (genome protein ID: 555728); Mt-WOX9= Medicago truncatula (accession number ABN09121); Vv-WOX9= Vitis vinifera (accession number CAO66373); At-WOX9-STIP= Arabidopsis thaliana Stimpy (accession number NP_180944); At-WOX8-STIPL= Arabidopsis thaliana Stimpy-like (accession number NP_199410). (2.73 MB JPG) [file pbio.0060288.sg004.jpg]

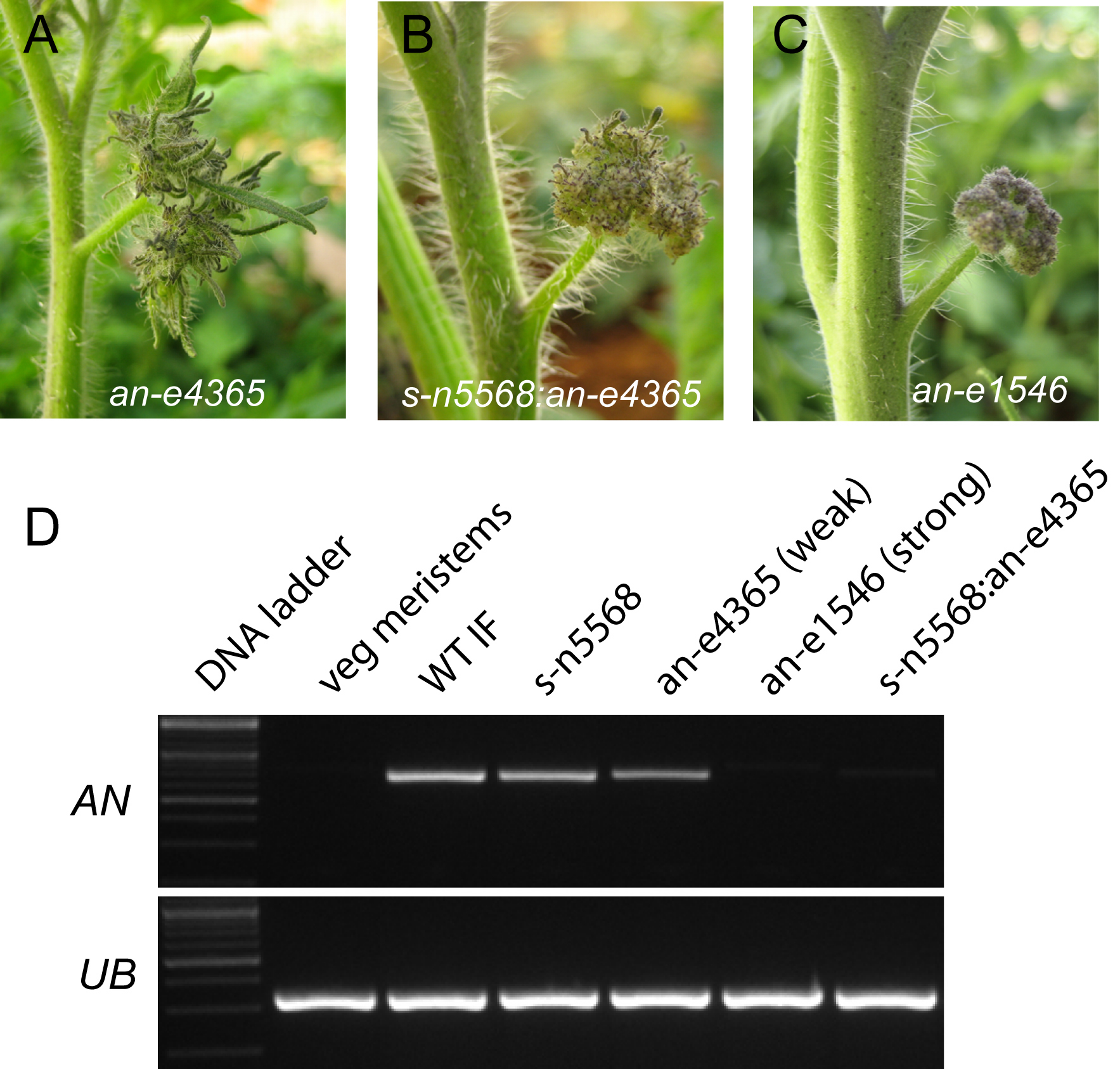

Supplement: Figure S5 — (A) Young branching inflorescence of a weak an allele (an-e4365) having sepals and carpelloid tissue, but lacking petals and stamens. (B) Double mutant of an-e4365 with s-n5568 showing an enhanced inflorescence phenotype having stronger floral organ defects resembling strong alleles of an, like an-e1546 shown in (C). (D) RT-PCR of AN expression on single and double mutants. The weak allele an-e4365 shows a modest reduction in AN expression relative to WT inflorescences. The strong allele an-e1546 has little or no AN expression, and a similar loss of expression is recapitulated in the enhanced double mutants s-n5568:an-e4365. This suggests either that S has a transcriptional regulatory role on AN or that the double mutant arrests at a developmental stage lacking early FM identity, and therefore does not express AN at substantial levels. (1.16 MB JPG) [file pbio.0060288.sg005.jpg]

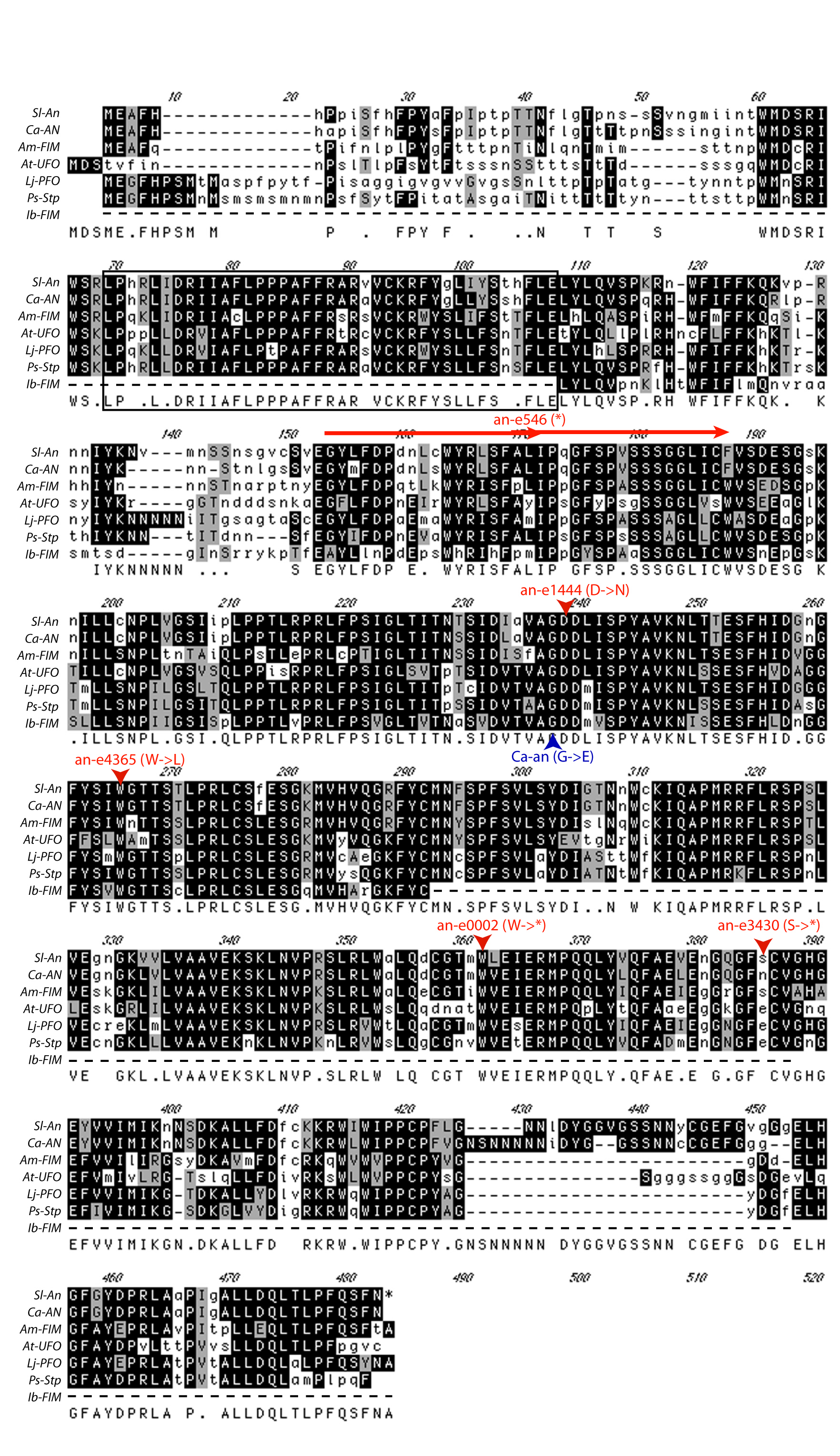

Supplement: Figure S6 — Highly conserved amino acids are shaded in black, and different amino acids of the same group are shaded in gray. The consensus sequence is shown below the alignment. Dashes denote gaps that were introduced to optimize the alignment. The F-box domain is boxed. The mutations for five alleles of an causing frame-shifts or amino acid changes are indicated above the alignment in red font. Red arrows for an-e1546 indicate the site of a tandem duplication. The pepper an (Ca-an) mutation is shown in blue below the alignment preceding an-e1444. Am-FIM= Antirrhinum majus FIMBRIATA (FIM, accession number S71192); At-UFO= Arabidopsis thaliana UNUSUAL FLORAL ORGANS (UFO, accession number X89224); Lj-UFO= Lotus japonicus Proliferating floral organs (PFO, accession number AAN87351); Ps-UFO= Pisum sativum Stamina pistilloida (Stp, accession number AF004843); Impatiens balsamina FIMBRIATA (Imp-FIM, accession number AF047392). (2.28 MB JPG) [file pbio.0060288.sg006.jpg]

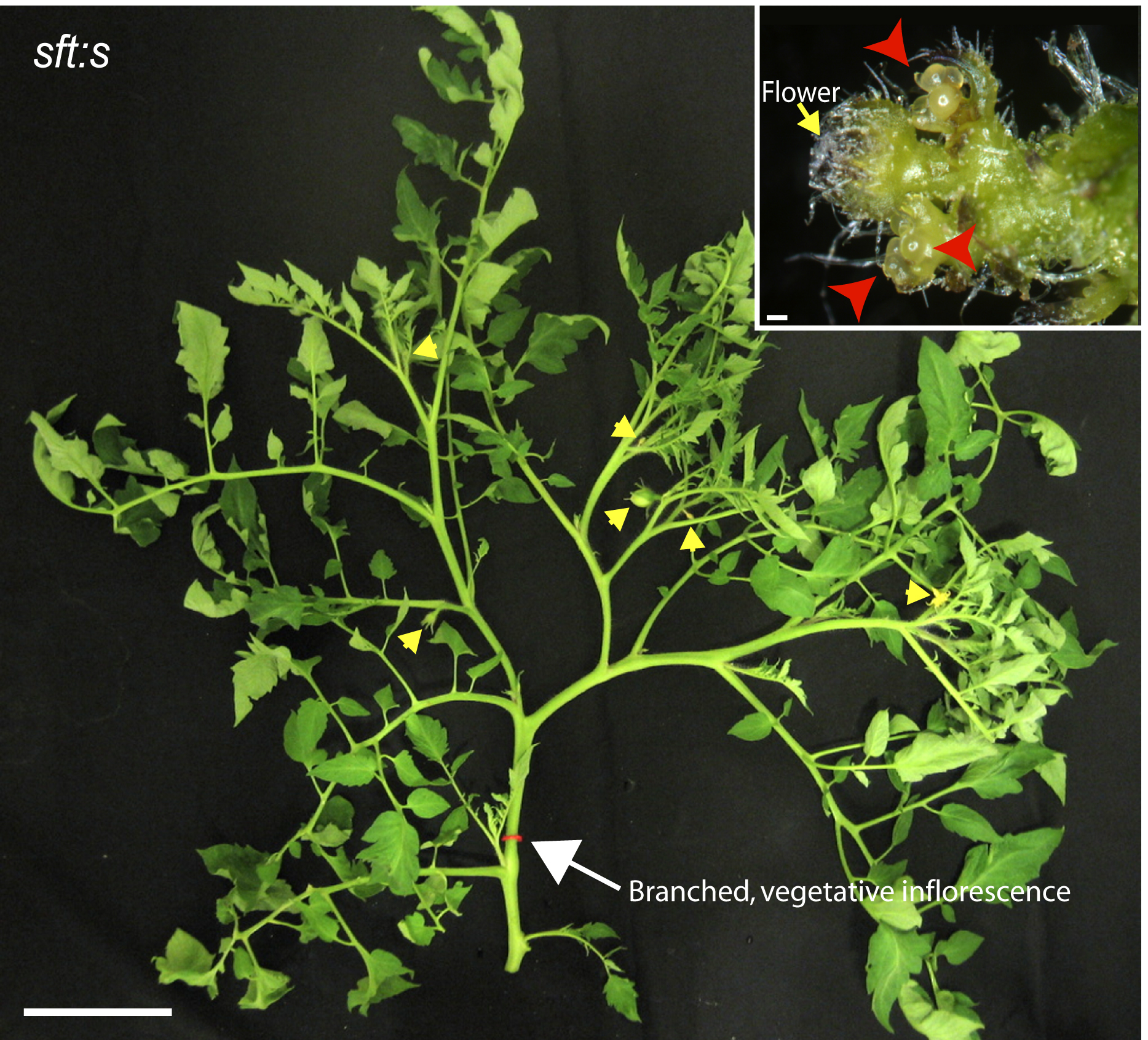

Supplement: Figure S7 — Double mutants between s and sft convert the single indeterminate vegetative inflorescence shoot of sft mutants to a highly branched vegetative inflorescence (red ring, white arrow) with dispersed single flowers (yellow arrowheads). SIM elaboration from a young developmental stage (inset) shows branches composed of leaflets and floral buds (red arrowheads) developing behind a single terminal flower (yellow arrow). These are the vegetative inflorescence branches that contribute to the architecture of mature double mutant inflorescences. Scale bars, 10 cm plant; 100 μm, inset. (1.95 MB JPG) [file pbio.0060288.sg007.jpg]

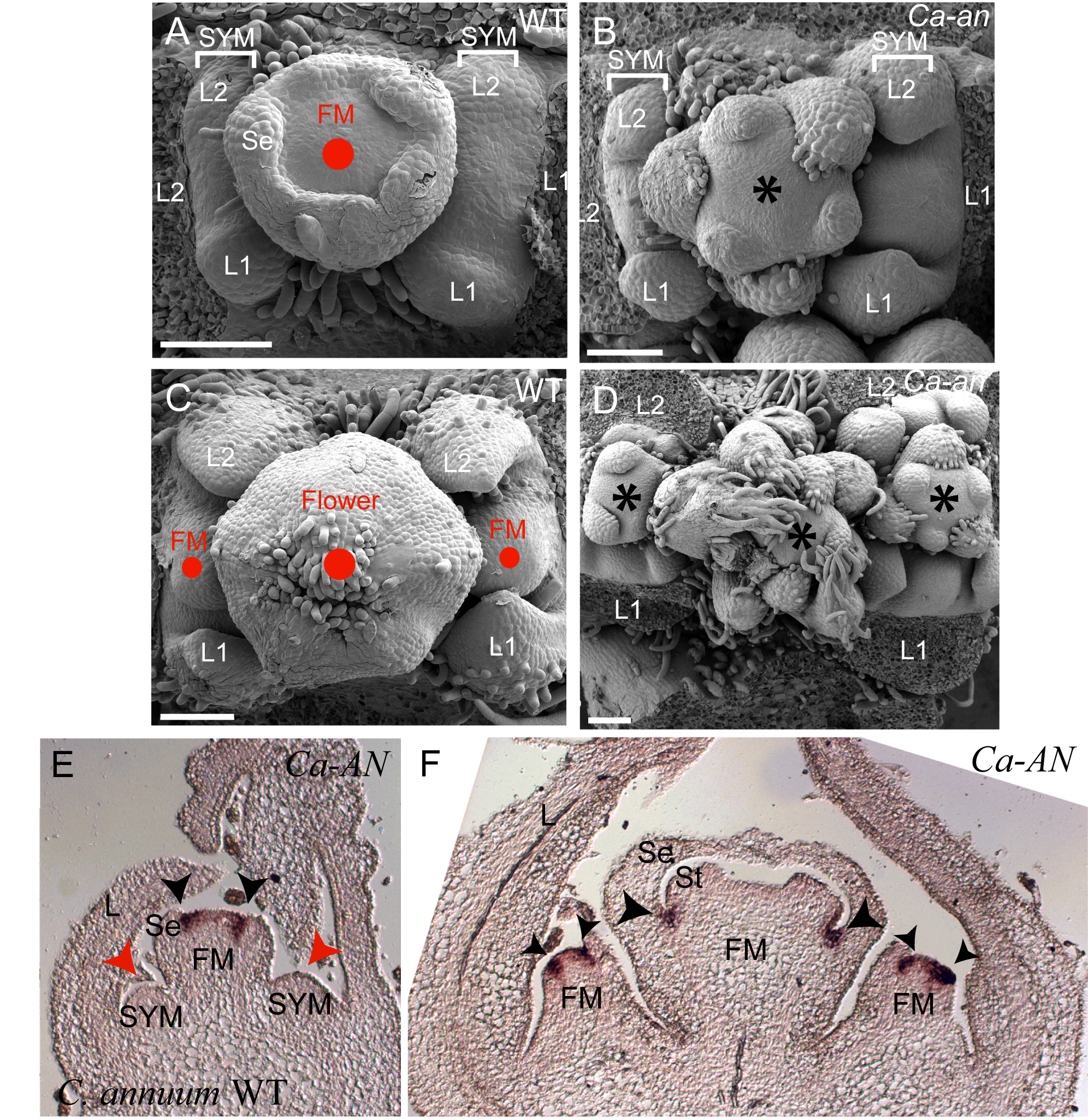

Supplement: Figure S8 — (A–D) Scanning electron micrographs showing two sympodial shoots (SYM) and distinct inflorescence stages from normal (A and C) and Ca-an mutants (B and D). Normal pepper produces a single floral meristem (FM, red dot) flanked by two SYMs, which are composed of two leaves and a single flower that terminates rapidly. Sympodial shoots arise reiteratively in the axils of each sympodial leaf and are released from apical dominance asymmetrically after floral termination, which is reflected in the slightly different developmental stages. Ca-an mutants produce an indeterminate shoot (asterisk) that repeatedly gives off lateral organs, which are, perhaps, modified sepals. Branching is infrequent at this early stage, and occurs more often in mature inflorescences or in the presence of modifiers, as shown in Figure 6F. (E and F) Detection of pepper Ca-AN expression by in situ hybridization. (E) Longitudinal section from a young inflorescence showing expression in the developing FM interior to sepal primordia (black arrows), but not in flanking SYMs (red arrows). (F) A later stage inflorescence with early stage FMs (left and right). The earliest expression of Ca-AN is observed in a ring (black arrows) between sepals (Se) and incipient petals. St= stamen; L= leaf; SYM= sympodial meristem; FM= floral meristem. Scale bars, 100 μm. (3.46 MB JPG) [file pbio.0060288.sg008.jpg]

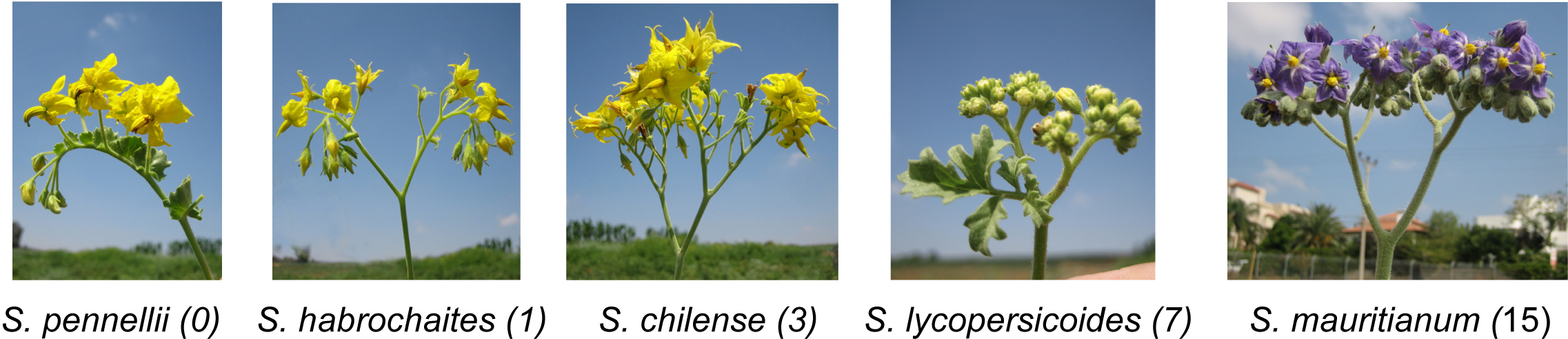

Supplement: Figure S9 — Quantification of inflorescence branching events from five species in the genus Solanum revealing that inflorescence branching varies widely between species and may have evolved multiple times. Numbers in parentheses indicate average number of branching events in each inflorescence. (674 KB JPG) [file pbio.0060288.sg009.jpg]
