# Supplementary material for: The Making of a Compound Inflorescence in Tomato and Related Nightshades
Source: PLoS Biol. 2008 Nov 18;6(11):e288. doi: 10.1371/journal.pbio.0060288 (PMC2586368; doi:10.1371/journal.pbio.0060288)
Supplement: Table S1 — Comparison of inflorescence branching events in three consecutive inflorescences from s mutant plants carrying a functional (SP+) or mutant (sp–) copy of SP, the tomato ortholog of TFL1. The first inflorescence after the transition to flowering (primary IF) showed a modest decrease in branching in s:sp double mutants (highlighted in gray). Interestingly, this effect was reversed in the two following inflorescences of the first (1st SYM IF) and second (2nd SYM IF) sympodial shoots of s:sp plants, which each showed more branching events than in s mutants alone (highlighted in gray). These effects are primarily indirect effects of sp mutants, whose primary change is on sympodial unit length (i.e., the number of leaves between sympodial units), which decreases progressively as sp plants mature [10]. This allows the initiation of both the first and second SYM IF in s:sp to occur earlier and, therefore, undergo more branching events compared to correspondingly younger inflorescences in s mutants alone. Eventually these younger inflorescences underwent a similar number of branching events, although more variation was introduced as inflorescences aged (unpublished data). (50 KB DOC) [file pbio.0060288.st001.doc]

**Table S1. Comparison of inflorescence branching events in three consecutive inflorescences from *s* mutant plants carrying a functional (SP+) or mutant (sp-) copy of *SP*, the tomato ortholog of *TFL1*.** The first inflorescence after the transition to flowering (primary IF) showed a modest decrease in branching in *s:sp* double mutants (highlighted in grey). Interestingly, this effect was reversed in the two following inflorescences of the first (1^st^ SYM IF) and second (2^nd^ SYM IF) sympodial shoots of *s:sp* plants, which each showed more branching events than in *s* mutants alone (highlighted in grey). These effects are primarily indirect effects of *sp* mutants, whose primary change is on sympodial unit length (i.e. the number of leaves between sympodial units), which decreases progressively as *sp* plants mature[2]. This allows the initiation of both the 1^st^ and 2^nd^ SYM IF in *s:sp* to occur earlier and, therefore, undergo more branching events compared to correspondingly younger inflorescences in *s* mutants alone. Eventually these younger inflorescences underwent a similar number of branching events, although more variation was introduced as inflorescences aged (not shown).
